# Effects of the subanesthetic dose of esketamine on postoperative sleep quality in patients undergoing modified radical mastectomy: a randomized, double-blind controlled trial

**DOI:** 10.3389/fmed.2025.1552934

**Published:** 2025-04-02

**Authors:** Ying Chen, Junchen He, Rong Huang, Zhuoqi Pan, Min Zhong, Wenxuan Zhang

**Affiliations:** ^1^Department of Anesthesiology, Guangdong Provincial Hospital of Chinese Medicine (The Second Affiliated Hospital of Guangzhou University of Chinese Medicine), Guangzhou, China; ^2^The Second Clinical Medical College of Guangzhou University of Chinese Medicine, Guangzhou, China

**Keywords:** esketamine, breast cancer, postoperative sleep disturbance, modified radical mastectomy, recovery

## Abstract

**Background:**

Breast cancer is the most common malignant tumor among women worldwide. Surgical intervention is a critical component of treatment, yet the associated stress and anxiety can significantly disrupt postoperative sleep quality. Emerging evidences suggest that esketamine may offer benefits in alleviating emotional distress and enhancing sleep. The purpose of this study was to observe the effects of intraoperative subanesthetic dose of esketamine on the sleep of patients undergoing modified radical mastectomy.

**Methods:**

This randomized, double-blind, controlled trial enrolled 145 female patients, who were randomly assigned to either the esketamine group (Group E, *n* = 72) or the control group (Group C, *n* = 73). Patients in Group E received esketamine (0.2 mg/kg loading dose, followed by 0.1 mg/kg/h infusion), while those in Group C received saline (0.2 mL/kg loading dose, followed by 0.1 mL/kg/h infusion). The primary outcome was the total score on the Richards-Campbell Sleep Questionnaire (RCSQ) measured on postoperative day (POD) 1. Secondary outcomes included recovery time, the incidence of postoperative adverse events and rescue analgesia, Visual Analogue Scale (VAS) pain scores, short-form McGill’s Pain Questionnaire (SF-MPQ) sensory and affective scores, and Pittsburgh Sleep Quality Index (PSQI) scores.

**Results:**

No significant differences were observed in the total RCSQ scores on POD 1 between Group E and Group C (median [interquartile range]: 46 [32–68] vs. 54 [40–71], *p* > 0.05). Recovery time was significantly longer in Group E compared to Group C (8 [5–11] vs. 6 [4–11] minutes; *p* = 0.02). There were no significant differences in the incidence of adverse events or remedial analgesia within 48 h postoperatively. Furthermore, no significant differences were observed between the groups in pain VAS scores, and SF-MPQ sensory or affective scores at 4, 24, and 48 h postoperatively. PSQI scores on POD 30 were not significantly different between the groups (*p* > 0.05).

**Conclusion:**

For female patients without pre-existing sleep disorders undergoing modified radical mastectomy, intraoperative subanesthetic esketamine may not significantly impact postoperative sleep quality but potentially contribute to a prolonged recovery time.

**Trial registration:**

This trial was registered at the Chinese Clinical Trial Registry on July 03, 2022 (https://www.chictr.org.cn; Registration number: ChiCTR2200061818).

## Background

Breast cancer, the third most common malignant tumor worldwide, is still prevalent and remains the leading cause of cancer-related deaths among women (1–3). Among available treatment options, modified radical mastectomy (MRM) is considered one of the most effective surgical interventions for breast cancer management (4). However, surgery imposes substantial physical and psychological stress, and anesthesia can disrupt the critical deep sleep phase. As a result, patients undergoing MRM often experience sleep disturbances, frequently accompanied by significant emotional disruptions (5–8).

Postoperative sleep disturbance (PSD) characterized by difficulties in sleep initiation and maintenance, poor sleep efficiency and quality, early awakening, and excessive daytime sleepiness is commonly observed in cancer patients after surgery (5, 9, 10). Approximately 64% of hospitalized patients suffer from sleep disruption and factors associated with the occurrence of PSD include postoperative pain, physical and mental dysfunction, fatigue, nausea, and vomiting (10). Beyond being a symptom of postoperative brain dysfunction, PSD is linked to severe health risks, including fatigue syndrome, metabolic disorders, and cardio-cerebrovascular diseases (11–17). These complications hinder recovery and prolong hospital stays, highlighting the importance of timely management and intervention (18).

Esketamine, a ketamine enantiomer with higher N-methyl-D-aspartic acid (NMDA) receptor affinity, serves as an adjunctive treatment for depression and improves patient outcomes (19–22). Beyond its antidepressant effects, esketamine also demonstrates efficacy in alleviating sleep disturbances, especially among patients with major depressive disorder (MDD) and insomnia (23). Esketamine has been shown to modulate sleep architecture and enhances sleep quality, potentially through its effects on glutamatergic neurotransmission within prefrontal-limbic circuits, anti-inflammatory pathways, and its interactions with the hypothalamic–pituitary–adrenal axis (24–26). Evidence from gynecological laparoscopy studies suggested that low doses of esketamine can effectively reduce PSD (27). Furthermore, previous research indicates that perioperative esketamine may help mitigate postoperative fatigue syndrome and enhance sleep quality in patients following laparoscopic gastric carcinoma resection (12).

Despite these encouraging findings, evidence on the effectiveness of esketamine in improving postoperative sleep quality following MRM remains limited. To balance efficacy and safety, we administered a subanesthetic dose of esketamine in this study, as supported by previous studies demonstrating its analgesic and anxiolytic benefits without inducing significant side effects (28). A randomized controlled trial was conducted using the Richards-Campbell Sleep Questionnaire (RCSQ) to evaluate the effects of perioperative administration of subanesthetic doses of esketamine on the sleep quality in patients undergoing MRM.

## Methods

### Ethics approval

This trial was approved by the Ethics Committee of Guangdong Provincial Hospital of Chinese Medicine (the Second Affiliated Hospital of Guangzhou University of Chinese Medicine) in May 2022. It was prospectively registered in the Chinese Clinical Trial Registry (ChiCTR2200061818) on July 03, 2022. Written informed consent was obtained from all participants before their enrollment. The study was conducted following the principles outlined in the Consolidated Standards of Reporting Trials (CONSORT) guideline.

### Design

This study was designed as a single-center, prospective, randomized, double-blind, placebo-controlled trial conducted at Guangdong Provincial Hospital of Chinese Medicine (the Second Affiliated Hospital of Guangzhou University of Chinese Medicine) between July 2022 and May 2024. Written informed consent was obtained from all participants prior to enrollment.

### Eligibility criteria

The inclusion criteria were as follows: (1) female patients aged 18–65 years; (2) American Society of Anesthesiologists (ASA) Classification I or II; (3) preoperative diagnosis of breast cancer scheduled for elective MRM under general anesthesia. (4) providing a written informed consent.

The exclusion criteria were as follows: (1) known allergy to any drugs used in the study protocol; (2) elevated intraocular pressure or severe preoperative liver and kidney dysfunction, cognitive impairment, psychosis, or epilepsy; (3) diagnosis of schizophrenia, mania, or any other psychiatric illness; (4) current use of sedative opioids, antipsychotic, or sleep-aid medication; (5) illiteracy or inability to understand the assessment scales used in this study.

### Randomization and preoperative management

Patients were randomly assigned in a 1:1 ratio to one of two groups using a computer-generated random sequence, with allocation concealed in sealed envelopes. Eligible participants were randomized to receive either esketamine or normal saline. An independent anesthesia nurse, not involved in the study, opened the envelope and prepared the study drugs according to the assigned group. The medications (esketamine or normal saline) were prepared in identical syringes with the same appearance and volume, labeled only with the study drug code and patient number. Both solutions were colorless and transparent, making them indistinguishable from one another. This study employed a double-blind design. Patients, anesthesiologists, surgeons, investigators responsible for data collection and outcome assessment, and statisticians remained blinded to group allocation throughout the study.

One day before surgery, preoperative visits were conducted by a blinded investigator. During these sessions, patients were provided with detailed explanations of the study protocol. Pittsburgh Sleep Quality Index (PSQI) was administered to assess baseline sleep quality and identify pre-existing sleep disturbances.

### Anesthesia protocol

All patients fasted for 8 h prior to surgery without receiving premedication. Upon arrival in the operating room, peripheral venous access was established, and sodium lactate Ringer’s solution was initiated. Patients were monitored according to the American Society of Anesthesiologists (ASA) monitoring standards, with the Narcotrend Index (NI) used throughout the procedure to assess the depth of anesthesia.

All patients received total intravenous anesthesia. Anesthesia induction involved the administration of sufentanil (0.3 μg/kg) and cisatracurium (0.2 mg/kg), along with continuous target-controlled infusions (TCI) of propofol (3–4 μg/mL) and remifentanil (2–4 ng/mL). The propofol TCI rate was adjusted to maintain a NI value of 37–64 (D0–D2, representing moderate to deep hypnosis), as per guidelines, to ensure adequate anesthesia while minimizing the risk of over-sedation or intraoperative awareness. Remifentanil infusion was titrated based on surgical stimulation and vital signs, maintaining fluctuations within 20% of baseline. Following endotracheal intubation, mechanical ventilation was initiated with a tidal volume of 6–8 mL/kg, a respiratory rate of 12 breaths per minute, and an inspired oxygen fraction of 50%. Ventilation parameters were adjusted to maintain peripheral oxygen saturation (SpO₂) ≥95% and end-tidal carbon dioxide (PetCO₂) between 35 and 45 mmHg. Anesthetic management was adjusted per the study protocol to maintain a NI value between 35 and 55, and the mean arterial pressure (MAP) within 20% of baseline values. Atropine (0.3 mg) was administered in cases of severe bradycardia (heart rate < 45 bpm), and aramine (0.3 mg) was given intravenously if systolic blood pressure (SBP) dropped below 80 mmHg or MAP fell below 60 mmHg. During surgery, cisatracurium (1–2 mg) and sufentanil (5–10 μg) were administered intermittently as needed, based on hemodynamic parameters. Approximately 30 min before the anticipated end of surgery, all patients received tropisetron (5 mg) to prevent postoperative nausea and vomiting (PONV), and parecoxib sodium (40 mg) for multimodal analgesia. Propofol and remifentanil infusions were discontinued at the end of skin suturing. After surgery, patients were extubated and transferred to the post-anesthesia care unit (PACU). All patients underwent standardized perioperative pain management. Rescue analgesia was administered as 50 mg intravenous flurbiprofen axetil when the VAS pain score exceeded 3. This threshold was predefined based on clinical guidelines and evidence indicating that a VAS score ≥ 3 is associated with significant discomfort and impaired recovery. The goal was to balance effective pain relief with minimizing excessive opioid use, which could adversely impact sleep quality and recovery. The investigators responsible for outcome assessment were blinded to the patients’ group assignments.

### Interventions

Patients in this study were assigned to one of two groups: the esketamine group (Group E) or the control group (Group C). Group E received an intravenous (IV) bolus of esketamine at 0.2 mg/kg following anesthesia induction, followed by a continuous infusion of esketamine at 0.1 mg/kg/h until the start of surgical sewing. Group C received an IV bolus of 0.9% saline at 0.2 mL/kg after anesthesia induction, followed by a saline infusion at 0.1 mL/kg/h until the same time point of group E.

### Sleep quality measurement

Perioperative sleep quality during the nighttime was assessed using the RCSQ scores, which is a validated tool for measuring patients’ self-reported sleep quality across five dimensions: sleep depth, sleep latency (time required to fall asleep), number of awakenings, sleep efficiency (percentage of time awake), and sleep quality during the previous night (29). Each dimension is rated on a 100-mm visual-analogue scale with 0 indicating the worst possible sleep and 100 indicating the best possible sleep. A total RCSQ score was calculated as a mean of the five individual scores. An additional item was included in the RCSQ to assess the impact of noise on sleep quality, scored using the same scale.

### Data collection

Demographic and clinical data were retrieved from the hospital’s electronic medical record system. Intraoperative parameters included drug consumption, test drug infusion time, anesthesia time, operation time, fluid infusion, blood loss, urine volume, and the incidence of hypotension and bradycardia. NI values and intraoperative vital signs, including SPO_2_, SBP, diastolic blood pressure (DBP), MAP, and heart rate (HR), were measured at the following time points: upon entering the operating room (T0), immediately after anesthesia induction (T1), immediately following intubation (T2), surgical incision (T3), end of surgery (T4).

Recovery time was defined as the period from the discontinuation of anesthetic administration to tracheal extubation, as documented by PACU nurses. Extubation was performed only after the patient met predefined criteria, which included regaining consciousness, the ability to follow simple commands, stable respiratory function with a respiratory rate (RR) >10 breaths per minute, and a tidal volume > 6 mL/kg. Postoperative pain intensity was evaluated using a VAS where 0 represented no pain and 10 indicated the worst imaginable pain. Sensory and affective dimensions of pain were assessed using the Short-Form McGill Pain Questionnaire (SF-MPQ) at 4-, 24-, and 48-h following surgery. Postoperative adverse events included nausea, vomiting, dizziness, headache, and delirium, and incidence of remedial analgesia were also recorded during the 48 h postoperative period.

### Outcome assessments

The primary outcome was the total RCSQ scores measured on postoperative day (POD) 1. RCSQ scores were also assessed on the preoperative day (Pre) and POD 2 to evaluate changes in sleep quality.

Secondary outcomes comprised recovery time, the incidence of postoperative adverse events and rescue analgesia within postoperative 48 h. Additionally, pain intensity was assessed using the VAS and sensory and affective scores were obtained from SF-MPQ at 4, 24, and 48 h post-surgery. PSQI scores were administered on the preoperative day and on POD 30 to assess long-term sleep quality.

### Statistical analysis

All statistical analyses were conducted using SPSS (version 26.0, IBM Corporation, Armonk, NY, United States). Continuous data were tested for normality and homogeneity using the Kolmogorov–Smirnov test and Levene’s test, respectively. Normally distributed data were expressed as mean ± standard deviation and compared using the independent two-sample *t*-test. Data with non-normal distributions were presented as median (interquartile range, IQR) and analyzed using the Mann–Whitney U test. Categorical variables were expressed as numbers (percentages, %) and analyzed using the chi-square test or Fisher’s exact test, as appropriate. For secondary outcomes including adverse events, between-group differences were assessed using the chi-square test or Fisher’s exact test as appropriate. Effect sizes for categorical variables were presented as risk ratios (RR) with their corresponding 95% confidence intervals (CI). An alpha of 0.05 was considered significant. A *p*-value <0.05 was considered statistically significant. GraphPad Prism 8.0 (GraphPad Software, San Diego, CA, United States) was used to generate all figures.

The sample size was determined based on the primary outcome. According to previous studies, the total RCSQ score on POD 1 for patients undergoing general surgery in the control group was 49 ± 20.5. A 10-point difference in the RCSQ score was considered clinically meaningful (5). A total of 66 patients per group was required to achieve 80% power with an alpha error of 0.05. Allowing for a 10% dropout rate, we planned to recruit 73 patients per group, resulting in a total sample size of 146 patients.

## Results

### Demographic characteristics and intraoperative data

**Figure 1:**
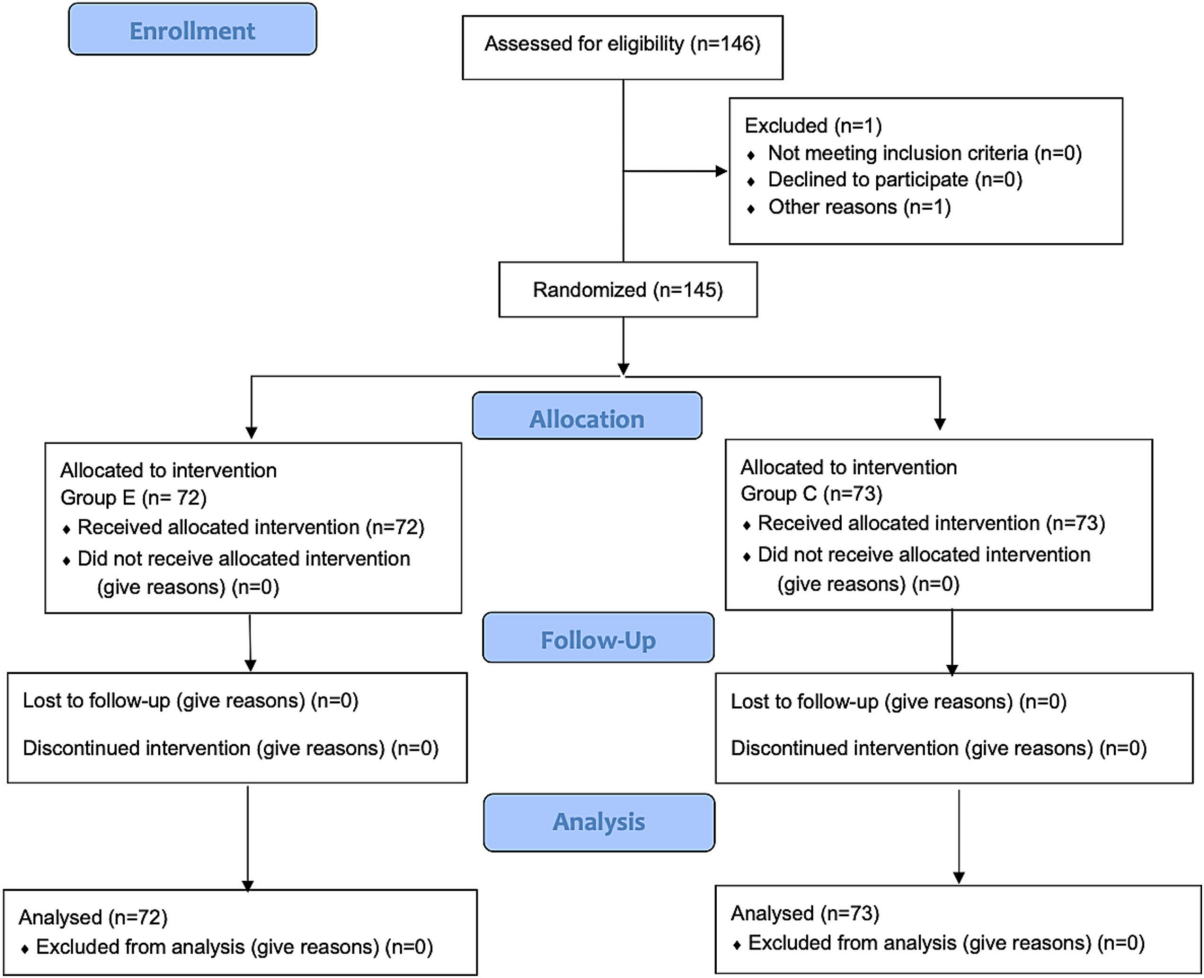
Consolidated standards of reporting trials (CONSORT) flow diagram.

**Figure 2:**
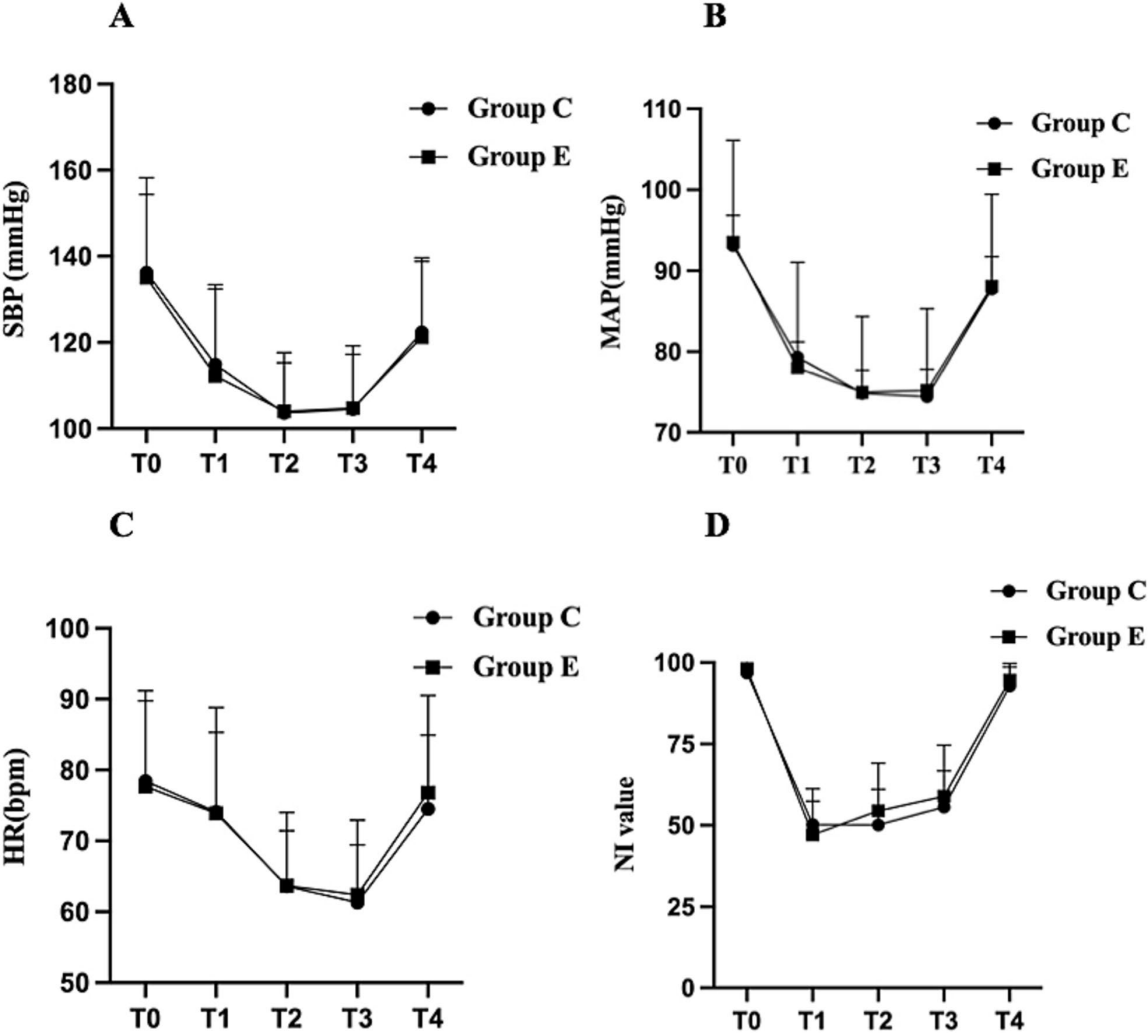
Changes in intraoperative hemodynamic parameters and NI values in the two groups. Data are expressed as mean ± standard deviation (SD). **(A)** SBP, **(B)** MAP, **(C)** HR, **(D)** NI value. The graphs display the mean ± SD of SBP, MAP, HR, and NI values at different time points. Intergroup comparisons between Group E and Group C at the same time points were analyzed using the independent two-sample *t*-test. SBP, systolic blood pressure; MAP, mean arterial pressure; HR, heart rate; NI, Narcotrend index. Time points: T0, entering the operating room; T1, immediately after anesthesia induction; T2, immediately after intubation; T3, surgical incision; T4, end of surgery.

A total of 146 female patients were assessed for eligibility, with one patient excluded based on the predefined exclusion criteria. Ultimately, 145 patients completed the study, with 72 in the esketamine group (Group E) and 73 in the control group (Group C). The CONSORT diagram is shown in Figure 1. Demographic characteristics, including age, body mass index (BMI), and ASA classification were collected for all participants. Intraoperative data were compared between the two groups, including intraoperative consumption of propofol, sufentanil, remifentanil, cisatracurium, and investigational product, as well as test drug infusion time, anesthesia time, operation time, fluids infusion, blood loss, urine volume, and the incidence of hypotension and severe bradycardia, were compared between the two groups. No significant differences were observed between the groups regarding baseline characteristics or intraoperative data (Table 1). Intraoperative hemodynamic measurements, including SBP, MAP, HR, and NI values did not show significant differences between the two groups (Figures 2A–D).

**Table 1:** Demographic and intraoperative data.

Variables	Group E (*n* = 72)	Group C (*n* = 73)	*p*-value^*^
Demographic			
Age (year)	48.30 ± 9.50	49.60 ± 9.20	0.37
BMI (kg/m^2^)	22.40(20.70–24.30)	23.10(21.10–25.00)	0.70
ASA classification (I/II)	12/60	13/60	0.27
Intraoperative data			
Propofol (mg)	720(585–910)	720(575–951)	0.70
Sufentanil (μg)	21.5(20.0–25.0)	20(20.0–25.0)	0.97
Remifentanil (mg)	0.50(0.40–0.69)	0.50(0.40–0.72)	0.86
Cisatracurium (mg)	12.0(10.0–14.0)	12.0(10.0–15.0)	0.35
Investigational product (mL)	18.5(17.0–21.8)	19.0(17.0–23.0)	0.48
Test drug infusion time (min)	81.5(67.0–107.3)	85.0(64.0–109.0)	0.90
Anesthesia time (min)	114.5(98.0–145.0)	118(95.0–140.5)	0.95
Operation time (min)	89.0(62.3–111.0)	90.0(69.0–112.5)	0.58
Fluids infusion (mL)	500(500–700)	500(500–725)	0.13
Blood loss (mL)	50.0(20.0–50.0)	25.0(16.3–50.0)	0.42
Urine volume (mL)	300(150–750)	300(225–425)	0.74
Hypotension^a^	3(4.2%)	2(2.7%)	0.99
Severe Bradycardia^b^	20(27.8%)	17(23.3%)	0.54
Recovery time (min)	8.0(5.0–11.0)	6.0(4.0–11.0)	0.02^*^

Data are presented as mean ± standard deviation, median (interquartile range, IQR), or *n* (%).

The *p*-values were calculated by the *t*-test, Mann–Whitney *U* test, or chi-square test.

^aSystolic blood pressure blood pressure less than 80 mmHg or mean arterial pressure less than 60 mmHg that required intravenous vasopressors, such as aramine.^

^bHeart rate of under 45 beats per minute that required intravenous atropine.^

*Statistically significant at *p*-value < 0.05 (two-tailed).

BMI, Body mass index; ASA, American Society of Anesthesiologists.

### Primary outcome

**Figure 3:**
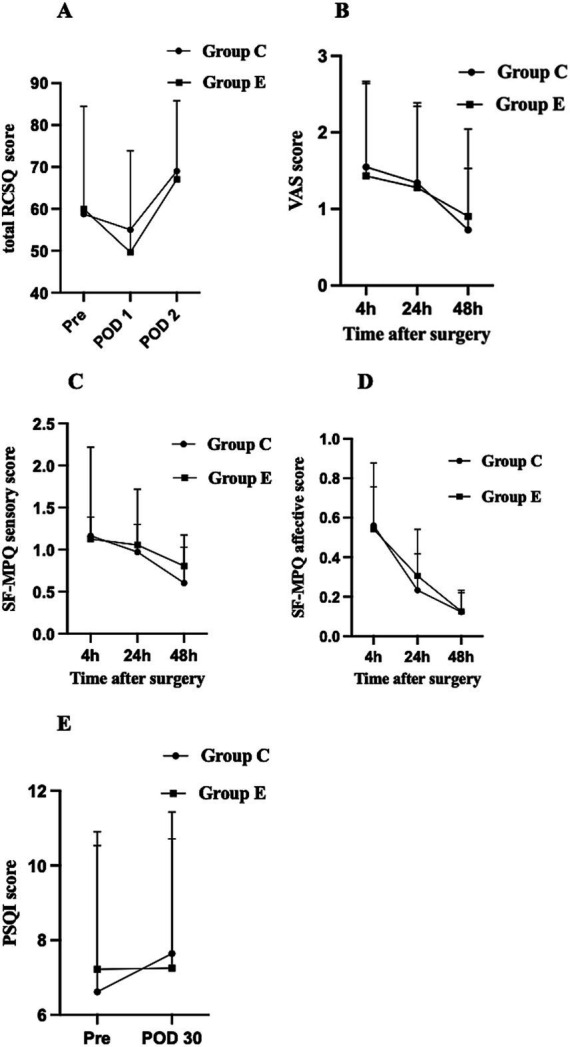
Perioperative changes in RCSQ score, VAS score, SF-MPQ sensory score, SF-MPQ affective score, and PSQI score among the groups. Data are presented as mean ± SD or median with IQR. **(A)** RCSQ score, **(B)** VAS score, **(C)** SF-MPQ sensory score, **(D)** SF-MPQ affective score, **(E)** PSQI score. Pre, the day before surgery; POD1, the first day after surgery; POD2, the second day after surgery; RCSQ, Richards-Campbell Sleep Questionnaire; PSQI, Pittsburgh Sleep Quality Index; SF-MPQ, short-form McGill’s Pain Questionnaire.

The distribution of RCSQ subscale scores for both groups during the perioperative period is presented in Table 2. There were no significant differences in the total RCSQ scores on POD 1 between group E and group C [46(32–68) vs. 54(40–71), *p* > 0.05]. Further, comparative analysis of the five subscale domains and noise disturbance scores revealed no statistically significant differences between groups during both preoperative and postoperative assessment periods (all *p* > 0.05). The perioperative changes in total RCSQ scores are illustrated in Figure 3A: on the preoperative day (Pre), total RCSQ scores were similar between groups; on POD 1, scores decreased in both groups but without significant intergroup differences; and on POD 2, scores showed a marked increase above baseline levels in both groups, with no significant differences between groups (*p* > 0.05).

**Table 2:** Distribution of RCSQ subscale scores on perioperative time.

RCSQ Item	Group E (*n* = 72)	Group C (*n* = 73)	*p*-value
Preoperative			
Sleep depth	70(43–80)	60(50–80)	0.36
Sleep latency	60(30–90)	60(35–80)	0.62
Awakenings	60(40–80)	60(40–80)	0.97
Returning to sleep	60(30–80)	60(30–80)	0.89
Sleep quality	70(40–80)	60(40–80)	0.43
Total score	64(40–80)	60(41–79)	0.67
Noise disturbance	80(63–100)	80(70–90)	0.75
POD 1			
Sleep depth	50(30–70)	50(40–70)	0.09
Sleep latency	50(30–80)	60(30–80)	0.19
Awakenings	45(30–60)	50(30–75)	0.26
Returning to sleep	50(30–60)	50(30–70)	0.09
Sleep quality	50(30–70)	60(40–80)	0.20
Total score	46(32–68)	54(40–71)	0.14
Noise disturbance	80(70–100)	80(70–90)	0.79
POD 2			
Sleep depth	70(50–80)	80(60–80)	0.42
Sleep latency	50(30–70)	50(34–70)	0.78
Awakenings	70(50–80)	70(60–80)	0.30
Returning to sleep	65(50–80)	70(50–80)	0.44
Sleep quality	70(60–80)	80(60–80)	0.79
Total score	70(51.5–80)	74(60–80)	0.45
Noise disturbance	80(70–100)	80(70–90)	0.84

Data are presented as median (interquartile range, IQR).

The *P*-values were calculated by the Mann–Whitney *U* test.

RCSQ, Richards-Campbell Sleep Questionnaire; POD, postoperative day.

### Secondary outcomes

Recovery time was significantly longer in Group E compared to Group C (8 [5–11] vs. 6 [4–11] minutes; *p* = 0.02) (Table 3). There were no significant differences between the groups in the incidence of adverse events, including nausea, vomiting, dizziness, headache, and delirium, nor in the need for rescue analgesia within the first 48 h post-surgery (*p* > 0.05) (Table 3). Additionally, no significant differences were found between the groups in VAS pain scores at 4, 24, and 48 h postoperatively (*p* > 0.05) (Figure 3B). Sensory and affective scores from the SF-MPQ at 4, 24, and 48 h postoperatively decreased over time but were similar between groups (*p* > 0.05) (Figures 3C,D). No statistically significant differences in PSQI scores were observed between the two groups on Pre and POD 30 (*p* > 0.05) (Figure 3E).

**Table 3:** Adverse events and rescue analgesic within postoperative 48 h.

Parameter	Group T (*n* = 72)	Group C (*n* = 73)	*p*-value	RR (95% CI)
Recovery period				
Nausea	1(1.4%)	2(2.7%)	1	1.01(0.97–1.06)
Vomiting	1(1.4%)	0(0%)	0.99	0.99(0.96–1.01)
Dizziness	11(15.3%)	4(5.5%)	0.06	0.90(0.80–1.00)
Headache	0(0%)	0(0%)	/	/
Delirium	0(0%)	0(0%)	/	/
Rescue analgesia	0(0%)	0(0%)	/	/
Postoperative 4 h				
Nausea	0(0%)	3(4.1%)	0.25	1.04(0.99–1.10)
Vomiting	0(0%)	1(1.4%)	1	1.01(0.99–1.04)
Dizziness	4(5.6%)	4(5.6%)	1	1.01(0.93–1.10)
Headache	0(0%)	1(1.4%)	1	1.01(0.99–1.04)
Delirium	0(0%)	0(0%)	/	/
Rescue analgesia	1(1.4%)	0(0%)	0.99	0.99(0.96–1.01)
Postoperative 24 h				
Nausea	2(2.8%)	3(4.1%)	1	1.01(0.95–1.08)
Vomiting	0(0%)	2(2.7%)	0.48	1.03(0.99–1.07)
Dizziness	2(2.8%)	1(1.4%)	0.99	0.99(0.94–1.03)
Headache	0(0%)	0(0%)	/	/
Delirium	0(0%)	0(0%)	/	/
Rescue analgesia	2(2.8%)	3(4.1%)	1	1.01(0.95–1.08)
Postoperative 48 h				
Nausea	0(0%)	0(0%)	/	/
Vomiting	0(0%)	0(0%)	/	/
Dizziness	1(1.4%)	0(0%)	0.99	0.99(0.96–1.01)
Headache	0(0%)	0(0%)	/	/
Delirium	0(0%)	0(0%)	/	/
Rescue analgesia	2(2.8%)	0(0%)	0.47	0.97(0.94–1.02)

Data are presented as *n* (%) or RR (95%CI).

The *P*-values were calculated by chi-square test.

RR, Risk Ratio; CI, confidence interval.

## Discussion

In this prospective double-blinded randomized trial, there was no statistically significant difference between groups regarding the total RCSQ score on POD 1, which suggests that intraoperative subanesthetic esketamine may not effectively improve early postoperative sleep quality in female patients undergoing MRM without pre-existing sleep disorders. Furthermore, no significant improvements were observed in VAS scores, the incidence of adverse events, SF-MPQ scores within the first 48 h postoperatively, or PSQI scores on POD 30. These results indicate that subanesthetic esketamine may not offer significant benefits in reducing postoperative pain, alleviating emotional distress.

However, intraoperative subanesthetic doses of esketamine were associated with a prolonged recovery time from general anesthesia in patients undergoing MRM, which is consistent with findings from previous studies. Zhang et al. (30) reported that subanesthetic doses of esketamine delayed anesthetic recovery in patients undergoing laparoscopic cholecystectomy. Similarly, Huang et al. (31) demonstrated that a combination of dexmedetomidine and esketamine provided enhanced sedation in a dose-dependent manner. Our findings align with previous studies, suggesting that esketamine may contribute to delayed recovery. A subanesthetic dose of esketamine as an adjuvant may enhance the sedative and analgesic effects of propofol and remifentanil during general anesthesia. However, intraoperative consumption of opioids and propofol was not reduced in the esketamine group, potentially contributing to the observed prolonged recovery duration. While the difference in recovery time may not be clinically meaningful in all settings, it could affect workflows in busy surgical centers or cases requiring rapid turnover. Within the framework of Enhanced Recovery After Surgery (ERAS) protocols, which prioritize minimizing recovery time and optimizing outcomes, these findings underscore the need for judicious use of esketamine. Strategies such as careful dose titration, lower infusion rates, or postoperative administration might mitigate this effect while preserving its analgesic and anxiolytic benefits.

Richards-Campbell Sleep Questionnaire is a simple and reliable questionnaire widely used to evaluate sleep quality during the previous night in hospitalized patients (29). It consists of five items assessing the perceived sleep depth, sleep latency, number of awakenings, efficiency, and sleep quality (32). In our study, we utilized the total RCSQ score to assess perioperative sleep quality and conducted further analyses of its five subcomponents. However, no significant differences were observed between the groups on POD 1. Similarly, Sun et al. (33) reported that continuous subanesthetic esketamine had no effect on improving postoperative sleep quality in patients undergoing laparoscopic radical resection for colorectal cancer. In contrast, Qiu et al. (27) demonstrated that intraoperative esketamine infusion during gynecological laparoscopic surgery improved the incidence of PSD. These inconsistent findings may be attributed to variations in the enrolled patient populations, esketamine dosing regimens, or surgical types. Qiu et al. (27) included patients with higher baseline levels of anxiety and sleep disturbances, who may be more responsive to the anxiolytic and sleep-enhancing effects of esketamine. MRM may not induce the same level of systemic inflammation or stress response as abdominal surgery, potentially reducing the observable effects of esketamine on sleep. Differences in dosing regimens could explain the varying results. Qiu et al. (27) used a higher dose of esketamine, which might have been more effective in modulating sleep-related pathways.

Brinck et al. (34) reported that intraoperative administration of high-dose esketamine (0.5 mg/kg loading, 0.6 mg/kg/h infusion) was associated with increased sedation and a higher incidence of drowsiness likely due to esketamine’s sedative properties. Even low dose esketamine can cause side effects, including psychiatric symptoms (25, 35, 36). Previous studies have also demonstrated delayed anesthetic recovery with subanesthetic doses of esketamine during laparoscopic cholecystectomy, aligning with our results (30). The dose-dependent side effects of esketamine highlight the importance of careful dosing and infusion rates (4, 37). We employed a subanesthetic bolus followed by continuous intravenous infusion to minimize side effects, aiming to achieve a favorable risk–benefit ratio by targeting patients most likely to benefit from the intervention.

Several factors may explain the underlying mechanisms for the lack of significant improvement in sleep quality with esketamine in our study. First, the subanesthetic dose of esketamine used was chosen to balance efficacy with safety. However, it is possible that this dose was insufficient to produce a meaningful effect on sleep architecture. Higher doses might be necessary to modulate NMDA receptor activity and downstream pathways that influence sleep quality. Future studies needed to explore dose–response relationships to identify the optimal dose for improving postoperative sleep. Second, the intraoperative administration of esketamine may have limited its impact on postoperative sleep quality. PSD are influenced by a complex interplay of factors, including pain, inflammation, and stress responses, which peak during the postoperative period. Administering esketamine during this critical window (either preoperative night or postoperatively) might yield different results.

Mounting studies have shown that preoperative depression, anxiety, and postoperative pain are significant risk factors for PSD (8, 17, 27). Depression and pain, in particular, have been shown to worsen sleep quality over time, contributing to higher PSQI scores (38). Studies in breast cancer patients suggest that depression significantly impacts sleep quality, with higher depressive symptoms correlating with worse preoperative sleep disturbances (38, 39). The factors affecting postoperative sleep quality in patients with preoperative sleep disorders are likely more complex. Previous studies primarily examined esketamine in high-risk PSD populations, whereas our study focused on a cohort with fewer psychiatric comorbidities and no preoperative sleep disorders, potentially explaining the limited effects observed (25, 27, 40). Esketamine may provide greater benefits for patients with higher preoperative anxiety, depression, or sleep disturbances, which will be the focus of our future research (41).

In addition to depression, postoperative pain is a key factor contributing to PSD, particularly in general surgical and orthopedic settings, where pain and sleep disturbances often interact (8, 42–45). Studies have shown that the highest incidence of sleep disturbances typically occurs after major surgical procedures, likely due to extensive tissue trauma and the overall severity of the patient’s condition (41). In contrast, patients undergoing MRM, which involves relatively less tissue trauma, tend to experience mild to moderate pain compared to those undergoing major abdominal surgeries. In our clinical practice, all patients received multimodal analgesia and the expertise and technical skills of the surgical team may have further minimized postoperative pain, explaining the low incidence of moderate-to-severe pain reported by patients in both groups. Adequate perioperative analgesia likely mitigated the influence of pain on postoperative sleep quality. Beyond pain, it is crucial to identify other factors associated with poor sleep quality to guide effective interventions for PSD (38). Non-pharmacologic approaches, including cognitive behavioral therapy focusing on sleep hygiene modification, mindfulness-based interventions, relaxation techniques, music therapy, and manual therapies, have proven effective in treating PSD (46, 47). A comprehensive, multidisciplinary approach that integrates non-pharmacologic and pharmacologic strategies is essential for optimizing recovery and improving the overall well-being of surgical patients.

This study has several limitations. First, while the monocentric design ensured methodological consistency, it may limit the generalizability of our findings. Future large-scale, multicenter, randomized, double-blind trials are needed to determine the optimal dosage, administration protocols, and efficacy of esketamine in improving postoperative sleep quality, particularly in high-risk populations. Second, the relatively small sample size may have limited the statistical power to detect differences in secondary outcomes, such as adverse events and pain scores. While the primary outcome of this study was adequately powered, the secondary analyses should be interpreted with caution due to the potential risk of Type II error. Third, the use of a low esketamine dose and the exclusion of patients with preoperative sleep disorders or depression may limit the applicability of our findings to broader populations. Recent evidence suggests that esketamine may benefit patients with insomnia or depression, highlighting the need for further research in these subgroups.

## Conclusion

In conclusion, our study suggests that for female patients without pre-existing sleep disorders undergoing MRM, intraoperative subanesthetic doses of esketamine may not improve postoperative sleep quality on POD 1. However, it may contribute to delayed postoperative recovery. Additionally, esketamine did not demonstrate significant benefits in reducing postoperative pain, emotional distress, enhancing psychological well-being. These findings indicate that intravenous subanesthetic doses of esketamine may have limited utility in this patient population. Further studies should explore appropriate doses that reduce the amount of anesthetic used, improve postoperative sleep quality, and do not affect postoperative awakening.

## Data Availability

The raw data supporting the conclusions of this article will be made available by the authors, without undue reservation.

## Glossary

RCSQ: Richards-Campbell Sleep Questionnaire
POD: Postoperative Day
VAS: Visual Analogue Scale
SF-MPQ: Short-Form McGill Pain Questionnaire
PSQI: Pittsburgh Sleep Quality Index
MRM: Modified Radical Mastectomy
PSD: Postoperative Sleep Disturbance
NMDA: *N*-methyl-d-Aspartic acid
MDD: Major Depressive Disorder
CONSORT: Consolidated Standards of Reporting Trials
ASA: American Society of Anesthesiologists
NI: Narcotrend Index
TCI: Target-Controlled Infusions
SpO₂: Peripheral Oxygen Saturation
PetCO₂: End-tidal Carbon Dioxide
MAP: Mean Arterial Pressure
SBP: Systolic Blood Pressure
PONV: Postoperative Nausea and Vomiting
PACU: Post-Anesthesia Care Unit
DBP: Diastolic Blood Pressure
HR: Heart Rate
RR: Respiratory Rate
IQR: Interquartile Range
BMI: Body Mass Index
